# Lessons from the Human Genome Project: Modesty, Honesty, and Realism

**DOI:** 10.3389/fgene.2017.00184

**Published:** 2017-11-23

**Authors:** Frank Emmert-Streib, Matthias Dehmer, Olli Yli-Harja

**Affiliations:** ^1^Predictive Medicine and Data Analytics Lab, Tampere University of Technology, Tampere, Finland; ^2^Institute of Biosciences and Medical Technology, Tampere University of Technology, Tampere, Finland; ^3^Department for Biomedical Computer Science and Mechatronics, University for Health Sciences, Medical Informatics and Technology (UMIT), Hall in Tyrol, Austria; ^4^College of Computer and Control Engineering, Nankai University, Tianjin, China; ^5^Institute for Intelligent Production, Faculty for Management, University of Applied Sciences Upper Austria, Steyr, Austria; ^6^Computational Systems Biology Lab, Tampere University of Technology, Tampere, Finland

**Keywords:** genomics, high-throuput technique, sequencing, medicine, bioinformatics and computational biology

The Human Genome Project (HGP) (Lander et al., 2001; Venter et al., 2001; Consortium, International Human Genome Sequencing, 2004) has been considered “the single most important project in biology and the biomedical sciences” (Collins et al., 1998) because it sequenced the human genome and enabled the development of a variety of different high-throughput “Omics” technologies (Ghosh and Poisson, 2009; Moreno-Risueno et al., 2010) on the genomics, transcriptomics, metabolomics and epigenomics level. More importantly, from the beginning of the HGP there were high hopes and expectations of its implications on pharmacogenomics and medicine (Collins, 1999; van Ommen et al., 1999; Collins and McKusick, 2001; Reiss, 2001).

When one evaluates the outcome of the HGP using criteria of the same category as for its promotion (see above), unfortunately, to date the wider impact on medicine has not been realized (Ball, 2010; Wade, 2010). Exemplarily, this is demonstrated by Figures 1A,B where for four major western countries, United States of America (USA), Australia (AUS), Germany (GER) and Finland (FIN), two global indices for characterizing the health state on the society level are shown. Figure 1A shows the life expectancy (LE) at birth and Figure 1B the number of deaths per 100, 000 persons from all cancer types, age-standardized (World Health Organization, 2015; OECD, Organisation for Economic Co-operation and Development, 2017). In both figures the vertical dashed line indicates the completion of the HGP as a milestone. From this time on the HGP could have made an impact on the society, respectively, its health state. Overall, there is a lack of any improvement following the year 2002, as can be seen from the continuation of the observable trends starting in the 1980s and 1990s in all four countries without an increase in the life expectancy or a decrease in mortality.

**Figure 1:**
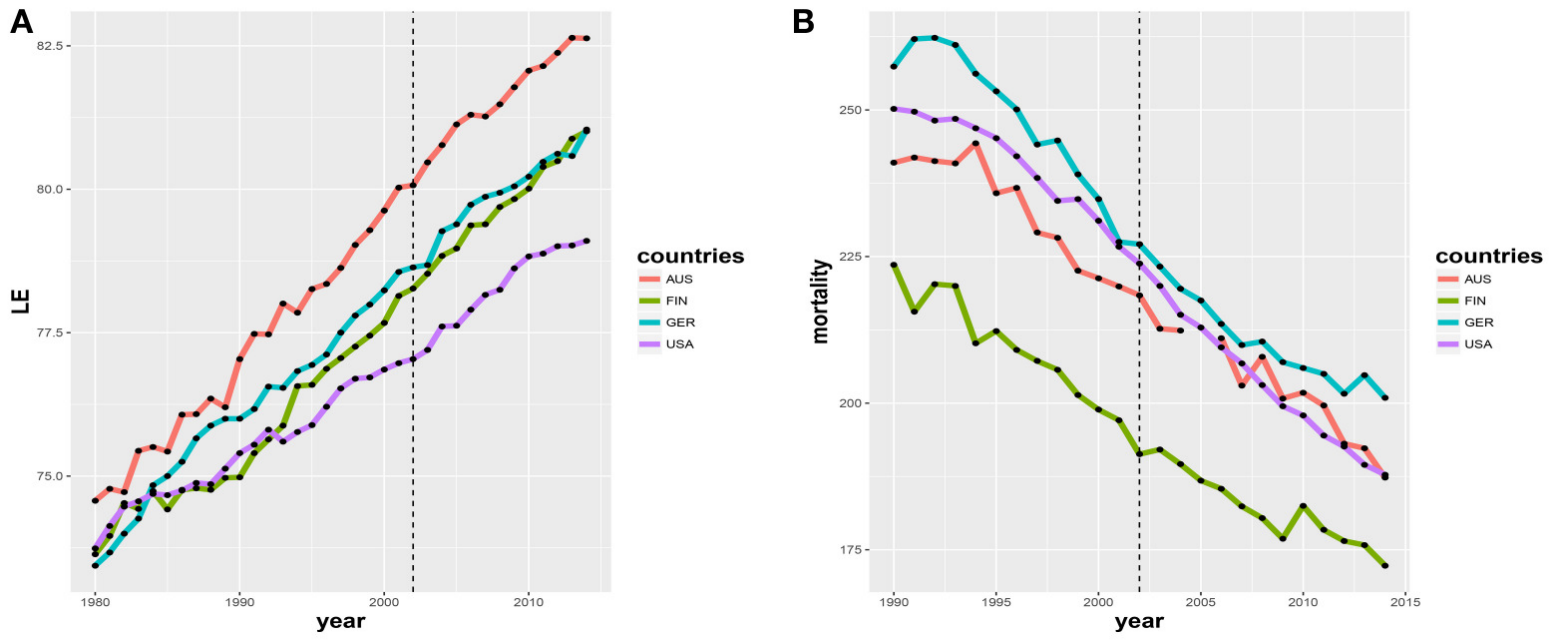
**(A)** Life expectancy (LE) at birth in the countries: United States of America (USA), Australia (AUS), Germany (GER) and Finland (FIN) (Human Mortality Database, 2012). **(B)** Deaths from cancer per 100, 000 persons over all types of cancer for the same four countries (World Health Organization, 2015; OECD, Organisation for Economic Co-operation and Development, 2017).

From these and similar observations, we think it is fair to state that over 15 years after the completion of the HGP there is no noticeable global impact on drug development and health. Given the estimated costs of this project with over $2.7 billion USD, there are valuable lessons to be learned for future projects.

First, we are of the opinion that expectations of research projects should be realistic. Since the introduction of the epigenetic landscape (Waddington, 1939) it is clear that there is a huge difference between individual genes and interactions between genes. Whereas the functional implications of the former is only clear in special cases, e.g., cystic fibrosis, the latter is the generic level of understanding for complex phenotypes and disorders like cancer, diabetes or schizophrenia. Hence, a project aiming for the individual gene level, as the HGP, can not lead to insights about complex phenotypes. It is clear that funding bodies like to advertise potential results of their projects as big as possible, but scientists should withstand such temptations because in retrospective, one can always assess the results to unveil a potential hype.

As a corollary from this, we are of the opinion that the expectations about “common” research projects, receiving funding in the order of magnitude of $10^5^ USD, need to be placed into perspective. That means realistic expectations of the impact from such projects on the society is about 10, 000 fold less than that of the HGP, because the HGP received more funding by a factor 10, 000. Hence, modesty in the formulation of goals and their potential impact should be appreciated.

Second, looking back in the history of science, revolutionary breakthroughs seem the result of individual scientists (with rather small groups and support) and not from multi-billion Dollar projects, like the HGP. For this reason, we suggest not to support such expensive projects in the future but, instead, distribute the funding among many small groups. In this way the likelihood of intellectual scientific progress is enlarged because many people work in parallel in a creative way on diverse problems. On the other hand, we acknowledge the need to develop biotechnology that is useful for conducting modern experiments. However, such projects need to be termed honestly and not disguised by marketing slogans in order to make them look more interesting than they in fact are.

Third, we would like to note that the HGP did not *formulate any particular biological hypothesis* to be tested but it's goal was to sequence the human genome with technology developed for this purpose. In other words the measurable outcome is the technology itself and as a specific result the human DNA. Put simply, the outcome is a general purpose tool, like a hammer. It can be used but it is up to you for what. Certainly, none would say that the Mona Lisa—the famous painting by Leonardo da Vinci—is attributed to the person who developed the hammer to hang the paining. In analogy it seem unreasonable to even compare the HGP to, e.g., the potential impact of the HPV vaccine to reduce mortality from cervical cancer, which was FDA approved in 2006 (Roden and Wu, 2006), based on the development of a drug resulting from dedicated experiments hypothesizing that this drug is beneficial in the treatment of cervical cancer. The honest assessment is that the HGP did not propose anything alike and hence anything that is a result of the application of its technologies should not be credited to it but the creativity of the researches that performed dedicated experiments to test specific hypothesis.

We are of the opinion that completed scientific projects of the size of the HGP need to undergo a rigorous assessment otherwise avoidable mistakes will be repeated in future projects. In general, we plead for more modesty, honesty and realism when proposing research projects and potential results because science is not like marketing where the ultimate goal is to sell a product by all means.

## Author contributions

FE-S conceived the study. All authors wrote the paper and approved the final version.

### Conflict of interest statement

The authors declare that the research was conducted in the absence of any commercial or financial relationships that could be construed as a potential conflict of interest.
